# Using Advanced Bioinformatics Tools to Identify Novel Therapeutic Candidates for Age-Related Macular Degeneration

**DOI:** 10.1167/tvst.11.8.10

**Published:** 2022-08-16

**Authors:** Urooba Nadeem, Bingqing Xie, Edward F. Xie, Mark D'Souza, David Dao, Dinanath Sulakhe, Dimitra Skondra

**Affiliations:** 1 Department of Pathology, University of Chicago, Chicago, IL, USA; 2 Department of Medicine, University of Chicago, IL, USA; 3 Chicago Medical School at Rosalind Franklin University of Medicine and Science, Chicago, IL, USA; 4 Center for Research Informatics, The University of Chicago, Chicago, IL, USA; 5 Department of Ophthalmology and Visual Science, University of Chicago, Chicago, IL, USA

**Keywords:** age-related macular degeneration, drug discovery, bioinformatics

## Abstract

**Purpose:**

Age-related macular degeneration (AMD) is the most common cause of aging-related blindness in the developing world. Although medications can slow progressive wet AMD, currently, no drugs to treat dry-AMD are available. We use a systems or in silico biology analysis to identify chemicals and drugs approved by the Food and Drug Administration for other indications that can be used to treat and prevent AMD.

**Methods:**

We queried National Center for Biotechnology Information to identify genes associated with AMD, wet AMD, dry AMD, intermediate AMD, and geographic atrophy to date. We combined genes from various AMD subtypes to reflect distinct stages of disease. Enrichment analysis using the ToppGene platform predicted molecules that can influence AMD genes. Compounds without clinical indications or with deleterious effects were manually filtered.

**Results:**

We identified several drug/chemical classes that can affect multiple genes involved in AMD. The drugs predicted from this analysis include antidiabetics, lipid-lowering agents, and antioxidants, which could theoretically be repurposed for AMD. Metformin was identified as the drug with the strongest association with wet AMD genes and is among the top candidates in all dry AMD subtypes. Curcumin, statins, and antioxidants are also among the top drugs correlating with AMD-risk genes.

**Conclusions:**

We use a systematic computational process to discover potential therapeutic targets for AMD. Our systematic and unbiased approach can be used to guide targeted preclinical/clinical studies for AMD and other ocular diseases.

**Translational Relevance:**

Advanced bioinformatics models identify novel chemicals and approved drug candidates that can be efficacious for different subtypes of AMD.

## Introduction

Age-related macular degeneration (AMD), an aging-related disease of the retina, is currently the leading cause of irreversible vision loss in developed nations. A meta-analysis projects that the number of patients with AMD will increase to 288 million globally and 22 million in the United States by 2040.^1^ Clinically, AMD is classified on the basis of disease severity into early, intermediate, and advanced forms. Early and intermediate forms are defined by the size of drusen deposits, whereas the advanced form includes either geographic atrophy (GA) or choroidal neovascular lesion.^2^^,^^3^ The conventional histopathologic classification divides the disease into dry AMD and wet AMD based on the absence or presence of neovascularization, respectively. Advanced neovascular AMD is treated successfully by antivascular endothelial cell growth factor (anti-VEGF) antibodies^3^^,^^4^; however, up to one fourth of wet-AMD patients are unresponsive to anti-VEGF based treatments, and another one third of the initial responders become resistant to the drug after multiple dosages over time.^5^ Despite anti-VEGF treatment in wet AMD patients, the aging-related degeneration continues to progress, and the vision gains obtained from therapy during the first two years of the trial were not maintained at five years.^6^ For dry AMD, there is no effective treatment available at the moment.^7^ Age-Related Eye Disease Study (AREDS) trials determined that antioxidant micronutrient supplements can decrease the risk of progression to wet AMD in intermediate AMD patients but conferred no benefit in early dry AMD patients or toward geographic atrophy.^8^^,^^9^

Conceiving and developing a single new drug costs about $2 to $3 billion on average.^10^^,^^11^ Recently, there has been a spate of drugs that failed clinical trials for dry AMD.^12^ Despite targeting different biologic pathways, including complement pathway inhibition, visual cycle inhibition, and molecules altering the release of neurotrophic factors; none of the approaches were successful.^13^^,^^14^ The commonly accepted “one disease–one target–one drug” approach has proved inadequate for diseases with multifactorial causes such as AMD.^15^ Recently, the National Advisory Eye Council task force proposed an interdisciplinary, unbiased systems biology approach of integrating big data available from clinical registries, network medicine, and integrative-omics to expedite finding new therapeutic targets for dry AMD.^16^ Effective therapeutic options for AMD should aim to target multiple biologic pathways that will most likely differ between the early, intermediate, and late stages of disease.^16^ Systems medicine and network pharmacology use computational and bioinformatics models that can integrate information from multiple causal pathways that affect disease processes simultaneously.^17^^,^^18^ These models are a better depiction of how gene alteration within a large molecular system can lead to disease. In the past, this approach successfully identified potential therapeutic targets for refractory epilepsy,^19^ cervical cancer,^20^ different glioma subtypes,^21^ asthma,^22^ colorectal cancer,^23^ Alzheimer's disease,^24^^,^^25^ and diabetic retinopathy.^26^

Another benefit of using network medicine is that it can identify previously approved drugs and repurpose them for indications other than their originally approved ones.^10^^,^^11^^,^^27^^,^^28^ Drug repurposing is increasingly being pursued as an alternative method to discover novel drugs for clinical trials. In contrast to developing a new drug, the cost of repurposing a drug is $300 million (approximately tenfold lower). Furthermore, the risk of failure from a safety viewpoint is significantly lower as safety has been previously assessed in both preclinical animal models and humans. It takes 10 to 14 years to get a new drug to market compared to the 6.5 years to repurpose an old drug for a new indication,^11^ as the Phase I and Phase II clinical trials have previously been performed. The field of AMD therapeutics is not foreign to drug repurposing; for instance, high-dosage atorvastatin, a lipid-lowering medication, showed benefit in high-risk AMD patients with regression of lipid deposits and improvement in visual acuity.^29^ A recent report found that metformin, an antidiabetic drug, is associated with reduced odds of developing AMD using big datasets.^30^

To our knowledge, no prior efforts have been made to predict potential drugs and chemicals for AMD via a systems medicine approach. Using a network-centric method, from all the described genes in AMD to date, we hypothesized that novel chemicals and known drugs may be identified.

## Methods

### Literature Search and Data Extraction

We queried the studies deposited in the National Center for Biotechnology Information database (https://www.ncbi.nlm.nih.gov/gene/) to compile a comprehensive list of genes described in AMD and subtypes of AMD-wet AMD, dry AMD, intermediate AMD, and geographic atrophy (GA) based on the well-accepted classification of different disease types.^2^ The collection of genes was performed according to the method described in previous studies.^31^^,^^32^

We also combined the genes involved in the following groups: intermediate and dry AMD; and intermediate AMD, dry AMD, and GA. The aim of combining the genes involved in these select groups is to reflect genes that play a role at distinct stages of AMD.

Medical Subject Headings (MeSH) terms for AMD were also queried, but as the AMD MeSH tree included “Stargardt Disease” and “Vitelliform Macular Dystrophy,” which are distinct disorders from AMD, MeSH terms are not suitable to collate genes that play a role in AMD. Genes that are not associated with AMD like ATP-binding cassette, subfamily A, member 4 (ABCA4), elongation of very long-chain fatty acids protein 4 (*ELOVL*), and prominin-like protein 1 (*PROM1*) and bestrophin 1 (*BEST1*) are manually removed from the final lists.^33^^–^^35^ Interestingly, if these genes had been retained in the analysis, they would have been manually filtered after building the unique sub-networks for AMD.

We also reviewed the abstracts of initial publications and collected the genetic association studies of AMD. We narrowed our selection via focusing on the selected publications, which reported significant associations between genes and AMD. The number of false-positive findings are reduced by excluding the publications that reported negative or insignificant associations. We reviewed the full texts of the selected publications and ensured that the content supported the conclusions. The genes, which were reported to be significantly associated with different types of AMD in these studies, were selected for this study. Ethical approval was not needed because this study does not involve humans or animals.

### Discovering Potential AMD Therapeutic Targets via Enrichment Analysis

Based on the hypothesis that the drugs will more efficiently work on disease genes if they show a tighter connection, the level of association between each of the candidate AMD target gene and relevant drugs were assessed to identify potential AMD target drugs as previously described.^36^^,^^37^

Therefore drug compounds that are both exclusively and highly interacting with the curated AMD genes can be potential AMD therapeutic targets. The enrichment analysis can provide a list of over-represented drug compounds regarding the input genes against the chemical-gene association database.

**Figure 1.**
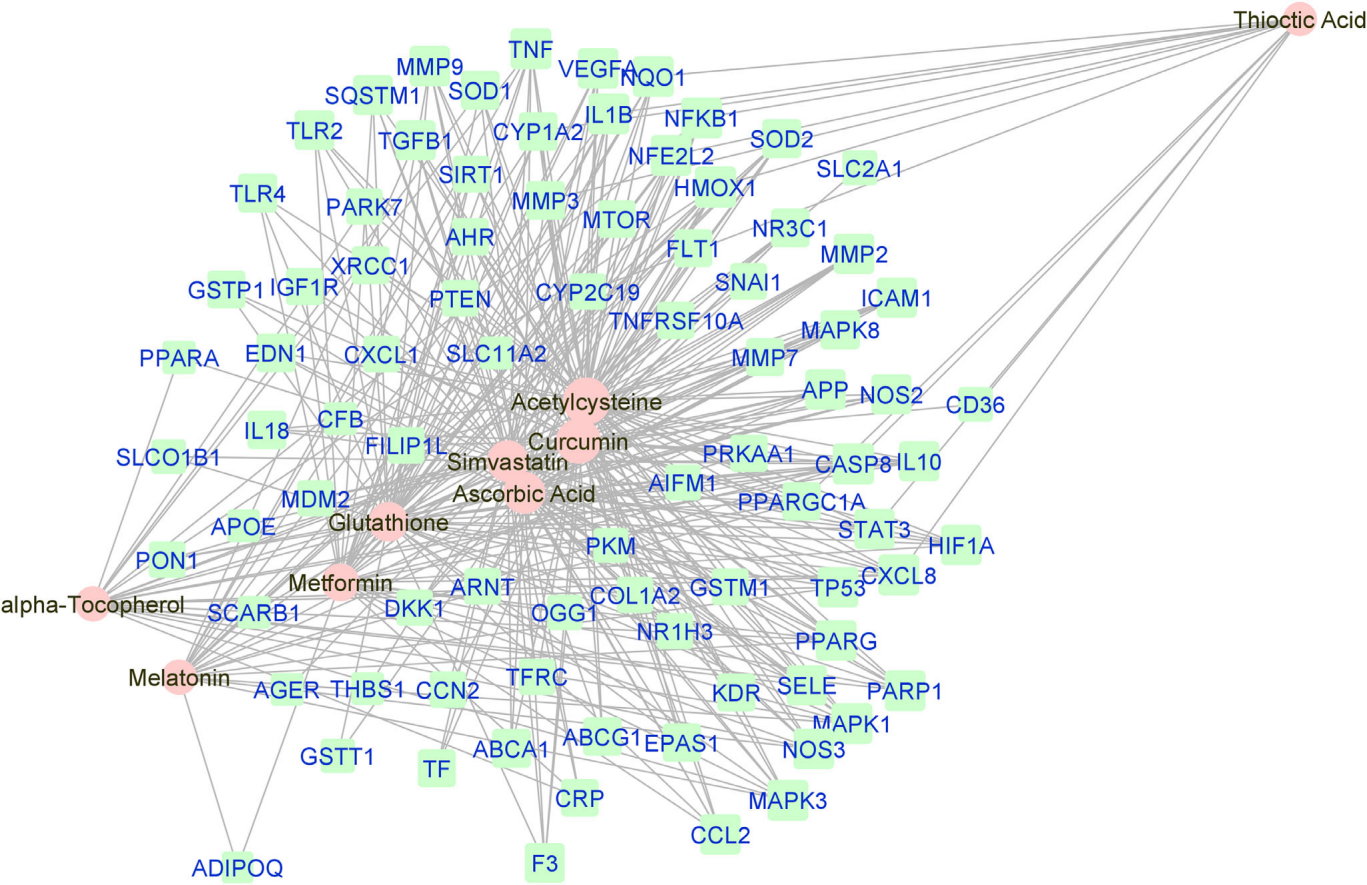
Force-directed graph of the interactions between drugs and genes related to (all) AMD. Nodes and edges are represented on the basis of centrality metrics analysis The drug nodes are colored in *pink* whereas the gene nodes are colored in *cyan*. Genes with fewer than three drug interactions are hidden for simplification.

Toppgene enrichment analysis against Pharmacome (Drug-Gene associations) is used for this analysis as it integrates drug annotations from five different sources including 77,146 total drug compounds^38^ for this analysis (Fig. 1). These sources include Broad Institute Connectivity Map (CMap, both up- and downregulation) Comparative Toxicogenomics Database (CTD), and Search Tool for Interactions of Chemicals (STITCH). All data sources contain both *curated* and *inferred* gene–chemical associations (Fig. 1).

The resulting drug gene interactions in our data are mainly from CTD and STITCH databases.^39^^,^^40^ CTD integrates data from curated scientific literature to describe chemical interactions with genes and proteins, whereas STITCH explores the known and predicted interactions of chemicals and protein by linking them to other chemicals and proteins by evidence derived from experiments, databases and the literature.^36^^,^^37^ The *P* value of each gene can be calculated by hypergeometric distribution as previously described using the equation below.^37^

P=1-∑i=0k-1MiN-Mn-iNi

In this equation, N is the total number of genes in the background distribution, M is the number of genes within that distribution that are annotated [either directly or indirectly] to the gene set of interest, n is the size of the list of genes of interest and k is the number of genes within that list that are annotated to the gene set. The background distribution by default is all the genes that have annotation. *P* values are also adjusted for multiple comparisons using the Benjamini-Hochberg Procedure to control the false discovery rate. In this analysis we considered drugs with false discovery rate adjusted *P* value <0.05.

Predicted drugs were also assessed for existing clinical relevance. We queried “drug target name AND age-related macular degeneration” and retrieved results from the clinical trial database (https://clinicaltrials.gov/ NIH, U.S. National Library of Medicine, Bethesda, MD, USA) (Table 1). We excluded querying existing compounds such as anti-VEGF agents and AREDS compounds from our results, which are already widely used clinically for AMD.

**Table 1.** Drug Targets That Have Corresponding Clinical Trials Based on clinicaltrial.gov Search Results

Drug Targets	Clinical Trial
Atorvastatin	NCT04735263
Metformin	NCT02684578
Curcumin	NCT04590196/NCT05062486
Antioxidant Formulations (ALA, NAC, Rutin)	NCT03919019^*^/NCT00893724^*^
Minocycline	NCT02564978

Anti-VEGF and AREDS drugs identified in our analysis have been excluded.

* Completed studies.

### Selection of Drugs/Chemicals Useful in AMD Based on Prior Knowledge

All the compounds deleterious to human health or compounds that cannot be used clinically such as particulate matter, ozone, and asbestos are manually removed. Redundant compounds from multiple databases are consolidated and the compound with the higher P value is retained.

### Reconstruction and Visualization of Networks

Manually curated genes for each AMD sub-category and the selected drugs enriched for those genes were used to reconstruct a drug-gene network. All the drug-gene interactions were extracted from the CTD database for combined curated genes and all selected drugs. For all disease subsets, we illustrate the binary association between gene and drug using Cytoscape.^41^ The drugs and genes are colored with red and green, respectively. The size of the nodes reflects the closeness centrality to highlight the potential hub nodes, where larger nodes tend to have higher closeness. The layout adopted is prefuse-directed layout on edge betweenness where a shorter edge indicates denser shortest path distribution between the two connecting nodes. The genes with less than three drug connections were further filtered from the network. The filtered network was then stratified by the genes and drugs corresponding to each AMD subcategory (Figs. 1^2^,^3^,^4^,^5^,^6^,^7^–8).

**Figure 2.**
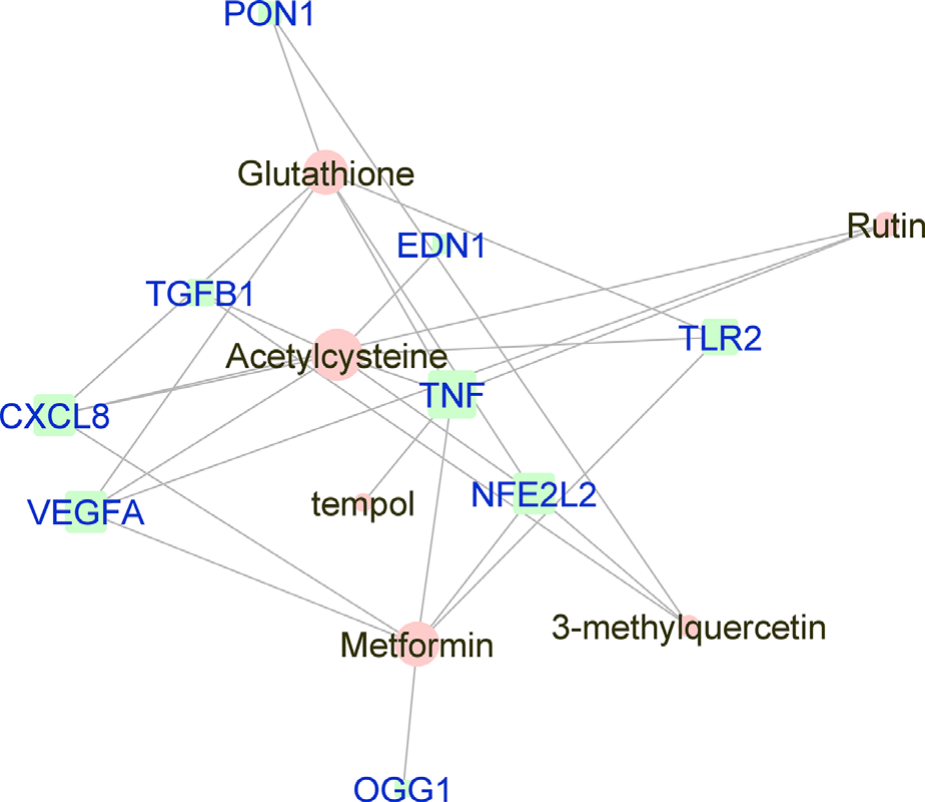
Force-directed graph of the interactions between drugs and genes related to wet-AMD. Nodes and edges are represented on the basis of centrality metrics analysis. The drug nodes are colored in *pink* whereas the gene nodes are colored in *cyan*. Genes with fewer than three drug interactions are hidden for simplification.

**Figure 3.**
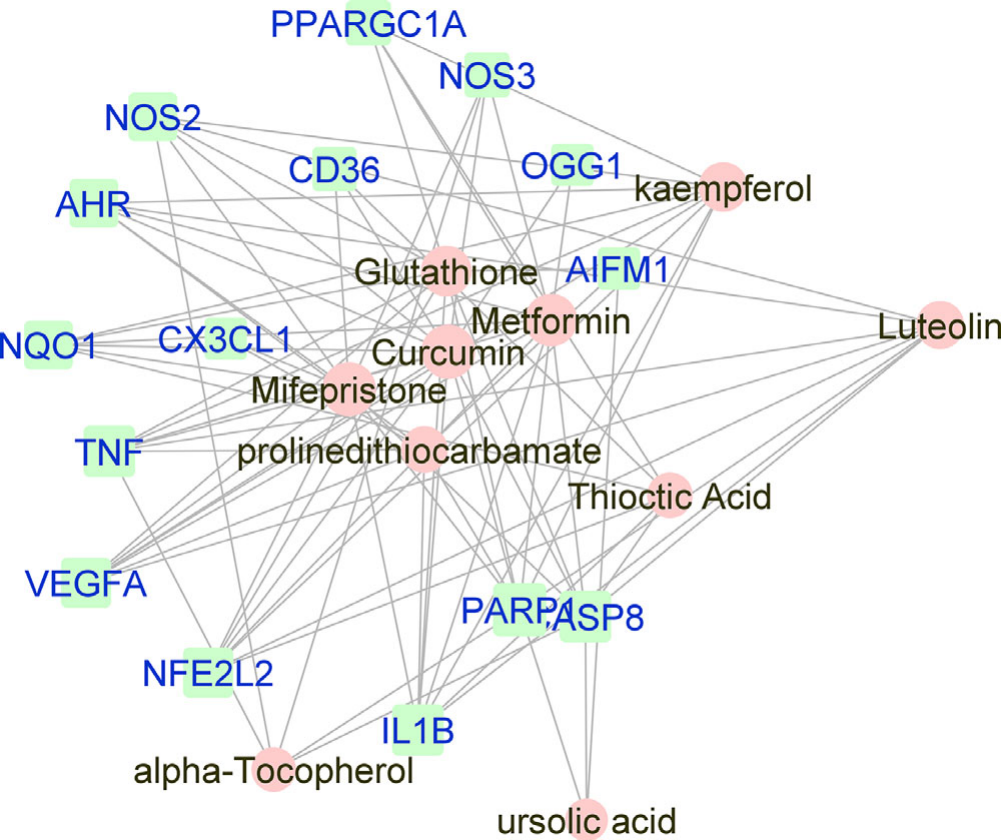
Force-directed graph of the interactions between drugs and genes related to dry-AMD. Nodes and edges are represented on the basis of centrality metrics analysis. The drug nodes are colored in *pink* whereas the gene nodes are colored in *cyan*. Genes with fewer than three drug interactions are hidden for simplification.

**Figure 4.**
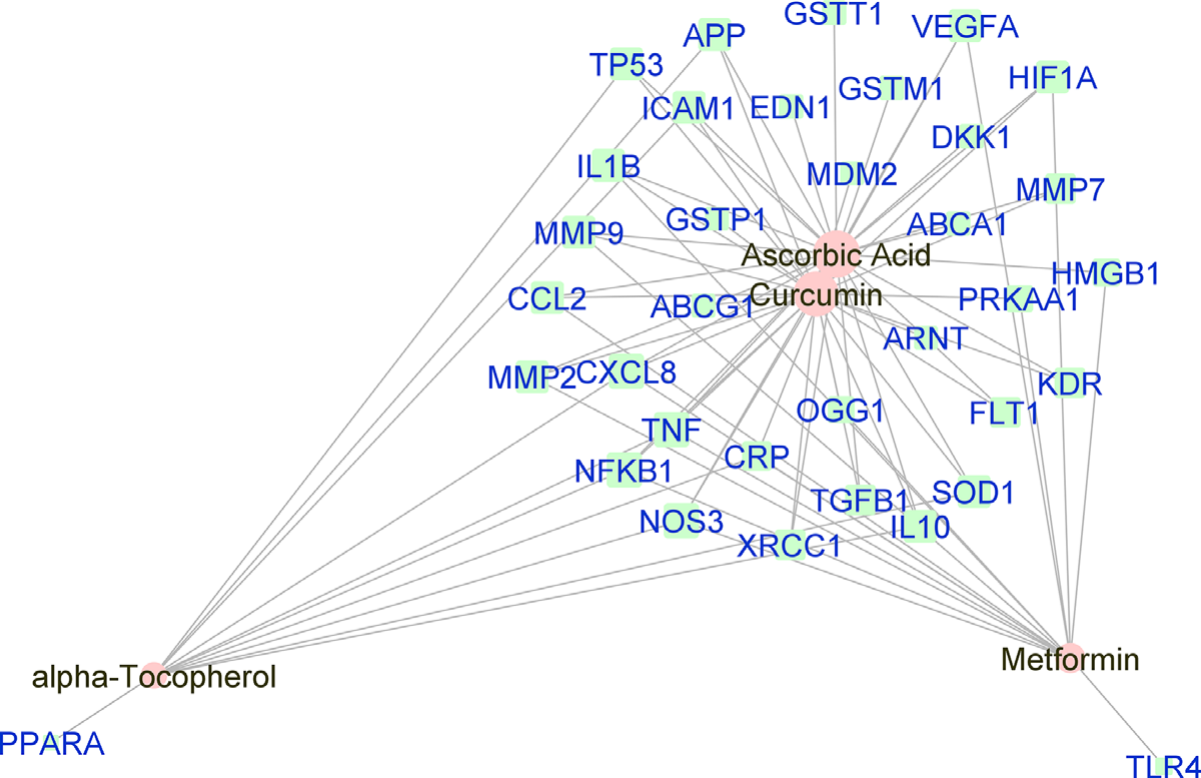
Force-directed graph of the interactions between drugs and genes related to intermediate-AMD. Nodes and edges are represented on the basis of centrality metrics analysis. The drug nodes are colored in *pink* whereas the gene nodes are colored in *cyan*. Genes with fewer than three drug interactions are hidden for simplification.

**Figure 5.**
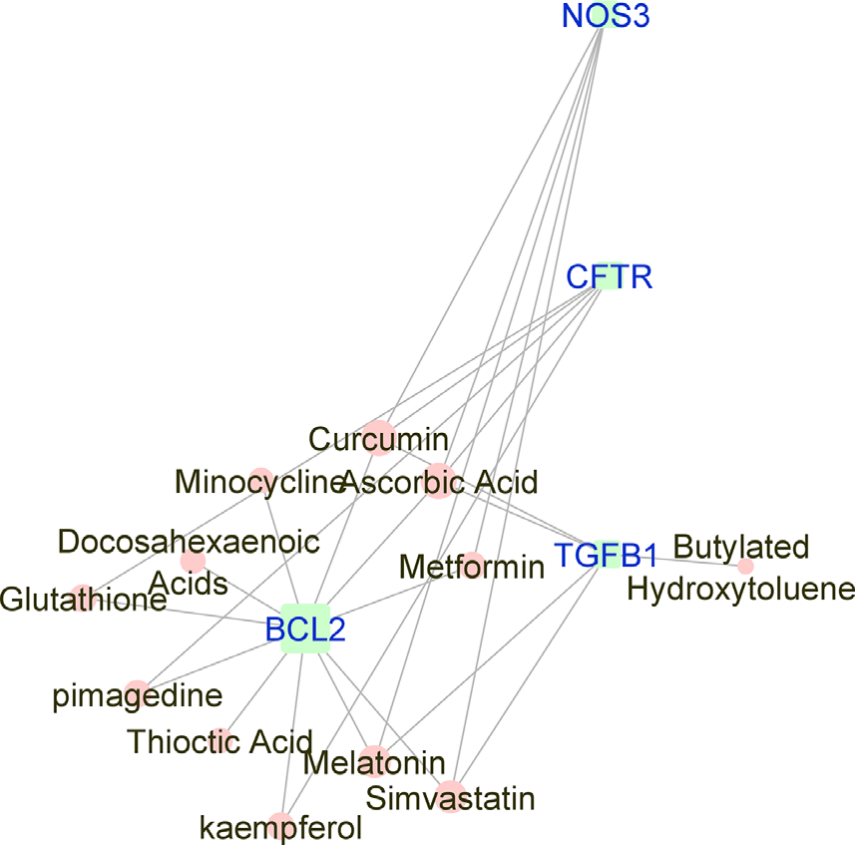
Force-directed graph of the interactions between drugs and genes related to geographic atrophy. Nodes and edges are represented on the basis of centrality metrics analysis. The drug nodes are colored in *pink* whereas the gene nodes are colored in *cyan*. Genes with fewer than three drug interactions are hidden for simplification.

**Figure 6.**
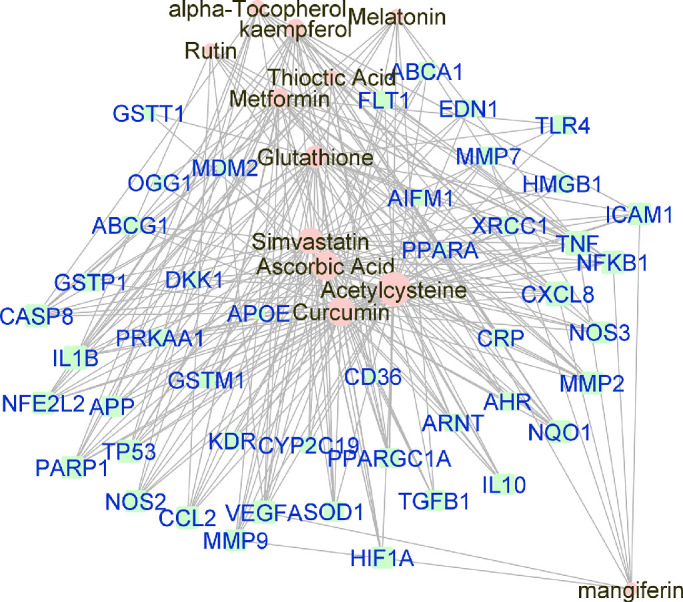
Force-directed graph of the interactions between drugs and genes related to combined intermediate and dry AMD. Nodes and edges are represented on the basis of centrality metrics analysis. The drug nodes are colored in *pink* whereas the gene nodes are colored in *cyan*. Genes with fewer than three drug interactions are hidden for simplification.

**Figure 7.**
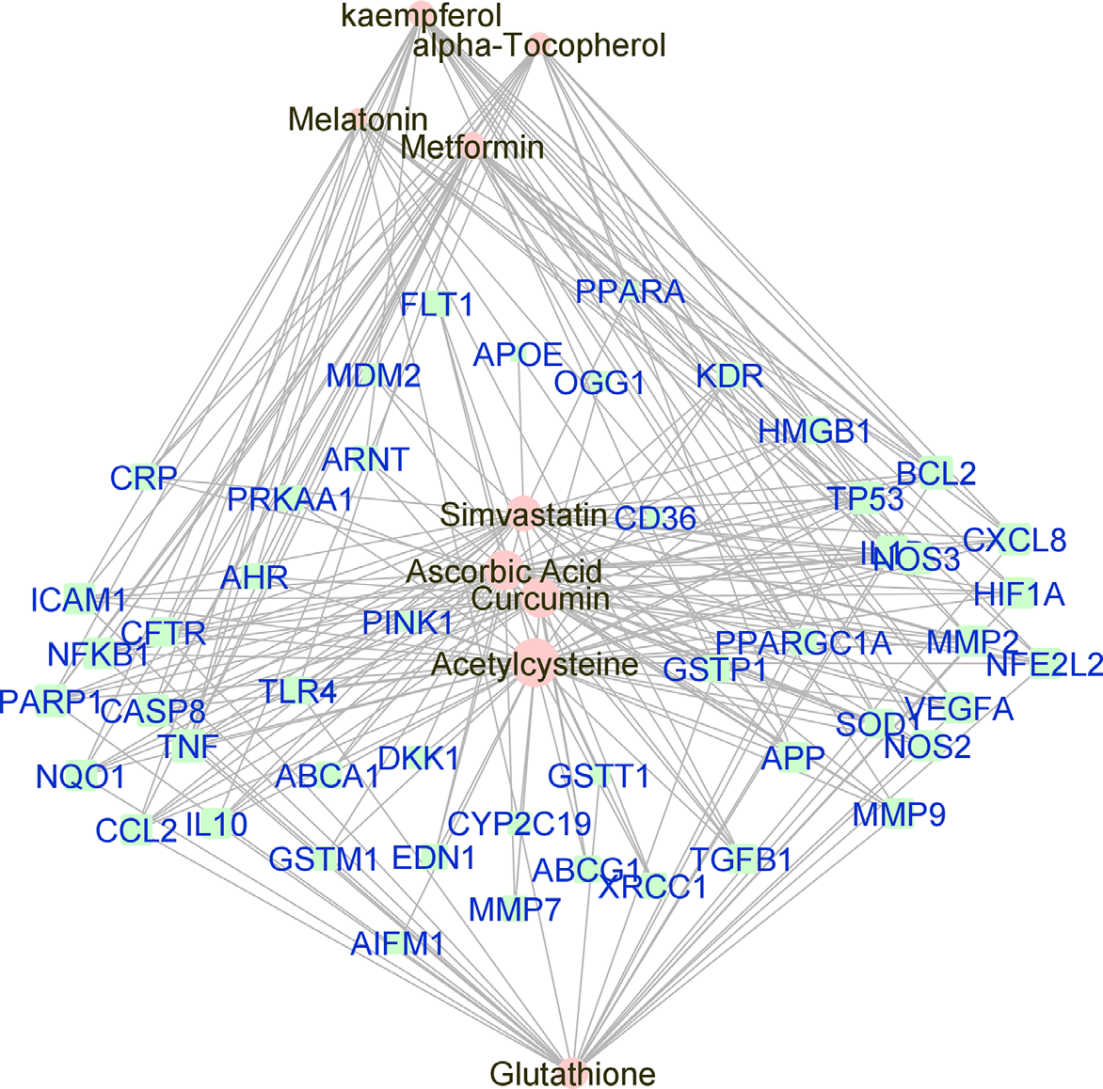
Force-directed graph of the interactions between drugs and genes related to combined geographic atrophy, dry, and intermediate AMD. Nodes and edges are represented on the basis of centrality metrics analysis. The drug nodes are colored in *pink* whereas the gene nodes are colored in *cyan*. Genes with fewer than three drug interactions are hidden for simplification.

**Figure 8.**
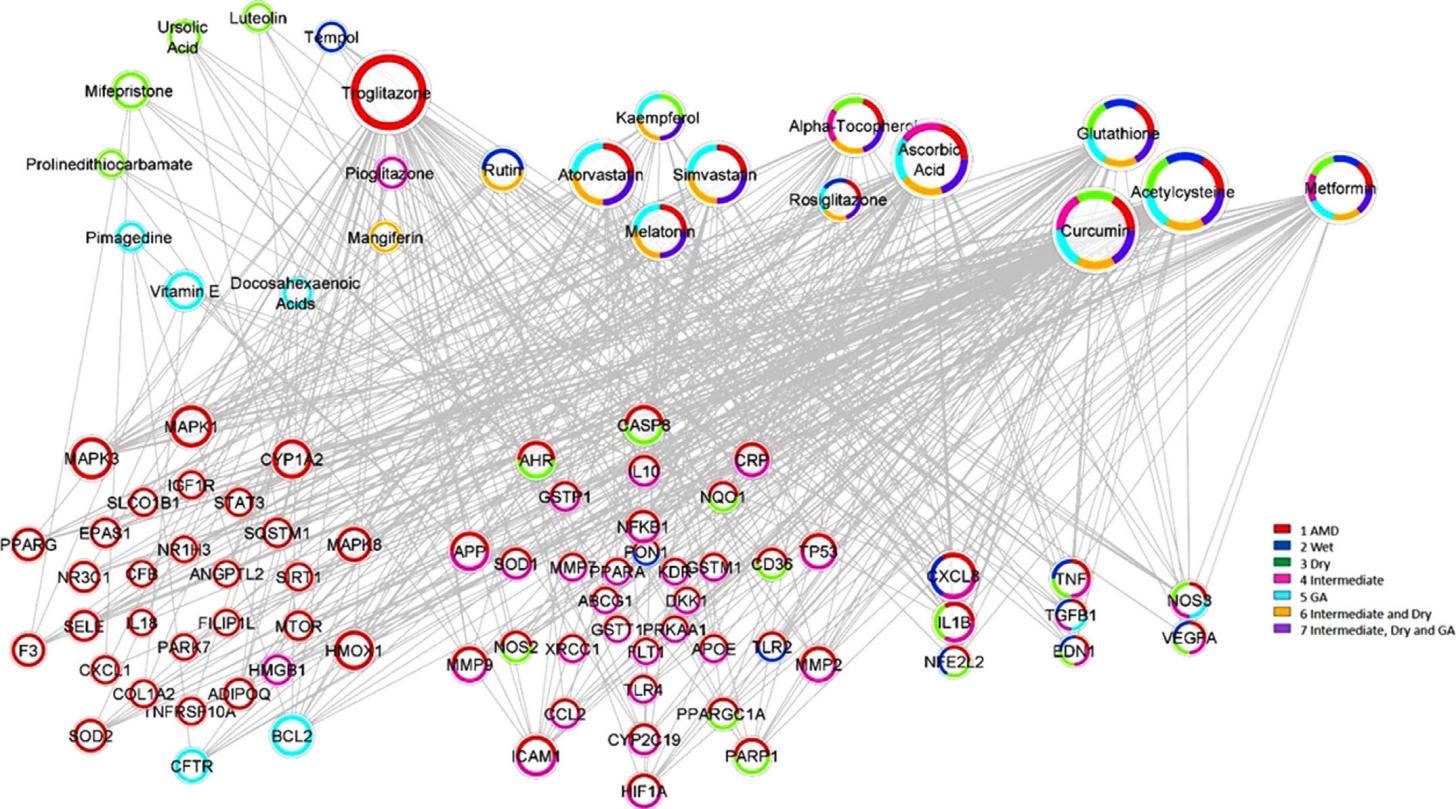
Force-directed graph for all drug-gene interactions. The node color of the drug is based on the gene enrichment from the AMD subcategories. The size of the node reflects the node degree. Nodes are grouped from left to right by order of association to the number of AMD subcategories. Note that for genes, the six and seven combined categories were derived from one to five categories.

**Figure 9.**
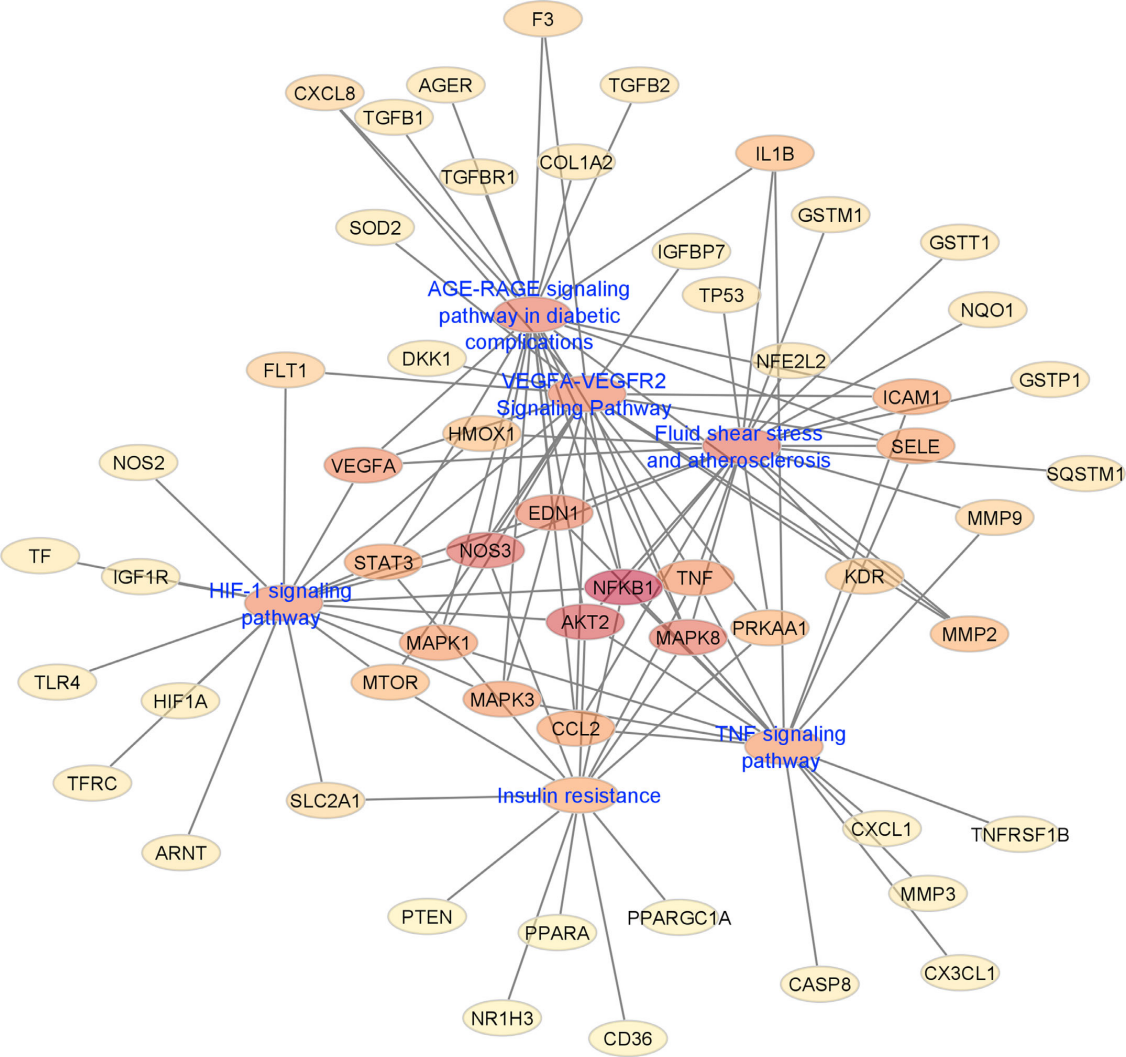
Force-directed graph for gene-pathway network. Nodes and edges are represented on the basis of centrality metrics analysis. The nodes in the network are colored by the centrality metric closeness from light yellow to dark red range highlighting the lower to higher closeness.

We also performed functional enrichment on gene lists from the seven AMD subcategories to create a gene-pathway network (Fig. 9). The enrichment analysis uses Lynx Enrichment tool on Gene Ontology (GO), disease, and pathway databases.^42^ We select six significant pathways (FDR adjusted *P* value < 7.22E-13) from the top enrichment results and construct the gene-pathway network from the general AMD category. We visualize and analyze the network using Cytoscape with the prefuse force directed layout on the centrality metric, edge betweenness.^43^ The nodes in the network are colored by the centrality metric closeness where light yellow to dark red range highlights the lower to higher closeness.

## Results

### Bioinformatics Analysis Reveals Chemicals and Drug Classes With Strong Associations With AMD Genes

The enrichment analysis with the curated AMD genes against the drug-gene database yields >1500 chemical compounds using FDR adjusted p-value cutoff of 0.05. We performed the manual selection based on the prior knowledge to remove the known deleterious compounds, for instance, particulate matter, ozone, and asbestos in the chemical compounds which resulted in a final list of 27 AMD-relevant chemical compounds. Total 886 interactions were extracted from the CTD database between the compounds and all 174 AMD genes to generate a highly connected bimodal network with a network density of 0.19. On average, a drug in the network is connecting to 33 genes which are about 19% number of the total AMD genes (Table 2).

**Table 2.** The Drug-Gene Network Properties Including Number of Drugs, Genes, Interactions, and the Network Density for Each AMD Subcategory

Condition	Drugs	Genes	Interactions	Density
AMD	9	165	392	0.26
Wet AMD	6	15	27	0.3
Dry AMD	10	27	85	0.31
Intermediate AMD	4	54	85	0.39
Geographic Atrophy	4	15	32	0.18
Combined intermediate and dry AMD	15	12	69	0.31
Combined intermediate, dry and geographic atrophy	9	77	252	0.36

The predicted drug classes that can be beneficial based on the risk-genes for AMD include antidiabetics agents, lipid-lowering agents, antioxidants, cardiovascular agents, and micronutrients. The antidiabetic agents identified in the study contain metformin (A10BA02), which may exhibit a glucose-lowering effect by inhibiting gluconeogenesis and increasing insulin sensitivity, and glipizide (A10BB07), which stimulates insulin release. Lipid-lowering agents include simvastatin (C10AA01) and atorvastatin (C10AA05) which are HMG-CoA reductase inhibitors that inhibit cholesterol synthesis. Interestingly, numerous micronutrients with antioxidative effects were identified including vitamins from the AREDS formula such as vitamin E (A11HA03) and vitamin C (A11GA01). Other antioxidative micronutrients include glutathione (V03AB32), which directly neutralizes reactive oxygen species (ROS), acetylcysteine (R05CB01), which replenishes glutathione reserves, and curcumin (V06), which may regulate NF-KB to exert an anti-inflammatory response in addition to scavenging ROS.^44^ Additionally, various cardiovascular agents such as aspirin (N02BA01) and enalapril isoproterenol (C09BA02), an ACE inhibitor, were also identified. Last, of note, investigational compounds such as MAP kinase inhibitor (SB203580) and MEK inhibitor (U0126) also strongly correlate with the AMD-risk genes although this class of drugs is poorly understood to date but may exhibit antineoplastic activities along with notable toxicities (Tables 3^4^,^5^,^6^,^7^,^8^–9).^45^

**Table 3.** Drugs Targeting All AMD Hub Genes Predicted by Toppgene Database

Filtered Position	Unfiltered Position	Name	Source	*P* Value	*Q* Value FDR B&H	Hit Count in Query List	Hit Count in Genome
1	1	Curcumin	CTD	1.52E-66	5.39E-62	94	851
2	2	Acetylcysteine	CTD	4.14E-59	1.47E-54	85	781
3	3	SB 203580	CTD	3.87E-58	1.38E-53	66	388
4	4	Ascorbic Acid	CTD	9.58E-55	3.40E-50	75	627
5	5	Simvastatin	CTD	1.54E-53	5.47E-49	72	581
6	6	Rosiglitazone	CTD	2.11E-48	7.49E-44	100	1571
7	7	Glutathione	CTD	2.32E-48	8.23E-44	56	339
8	19	Betamethasone	STITCH	4.69E-42	1.67E-37	87	1342
9	21	U 0126	CTD	1.07E-41	3.81E-37	56	444
10	23	Losartan	CTD	4.39E-40	1.56E-35	39	165
11	24	Melatonin	CTD	9.67E-40	3.43E-35	44	243
12	26	Diclofenac	CTD	5.17E-39	1.84E-34	59	570
13	31	Alpha-Tocopherol	CTD	3.11E-37	1.11E-32	37	164
14	33	Deferoxamine	CTD	1.83E-36	6.50E-32	38	186
15	34	Capsaicin	CTD	4.87E-36	1.73E-31	53	488
16	36	Troglitazone	CTD	3.41E-35	1.21E-30	79	1329
17	38	Enalapril	CTD	5.27E-35	1.87E-30	33	131
18	39	Aspirin	CTD	9.05E-35	3.22E-30	59	678
19	41	Thioctic Acid	CTD	1.95E-34	6.92E-30	35	163
20	45	Atorvastatin Calcium	CTD	1.13E-33	4.02E-29	36	186
21	48	Metformin	CTD	1.69E-33	6.02E-29	47	400
22	51	Prolinedithiocarbamate	CTD	2.30E-33	8.17E-29	25	59
23	60	Cobalt	CTD	4.21E-32	1.49E-27	40	277
24	64	Isoproterenol	CTD	2.62E-31	9.32E-27	66	1017
25	71	Naringin	CTD	2.03E-30	7.19E-26	31	146

**Table 4.** Drugs Targeting Wet-AMD Hub Genes Predicted by Toppgene Database

Filtered Position	Unfiltered Position	Name	Source	*P* Value	*Q* Value FDR B&H	Hit Count in Query List	Hit Count in Genome
1	3	Metformin	CTD	6.01E-12	1.44E-07	9	400
2	5	Carbocysteine	CTD	1.89E-11	3.47E-07	4	8
3	6	Hydrocortisone	CTD	2.94E-11	4.49E-07	7	167
4	11	Glutathione	CTD	8.36E-11	6.98E-07	8	339
5	12	Linoleic Acid	CTD	1.01E-10	7.76E-07	6	99
6	13	Rutin	CTD	1.45E-10	1.03E-06	6	105
7	15	Mevalonic Acid	CTD	2.04E-10	1.16E-06	6	111
8	16	Nifedipine	CTD	2.15E-10	1.16E-06	6	112
9	19	SB 203580	CTD	2.44E-10	1.16E-06	8	388
10	20	Diphenyleneiodonium	CTD	2.53E-10	1.16E-06	6	115
11	21	Ro 31-8220	CTD	2.78E-10	1.22E-06	5	48
12	27	3-methylquercetin	CTD	4.65E-10	1.58E-06	5	53
13	28	Methylprednisolone	CTD	5.08E-10	1.67E-06	6	129
14	32	Heparin	CTD	8.34E-10	2.39E-06	6	140
15	35	Eriodictyol	CTD	1.60E-09	3.95E-06	4	21
16	37	Chetomin	CTD	1.65E-09	3.95E-06	3	4
17	41	Candesartan	STITCH	2.01E-09	4.51E-06	6	162
18	42	Tempol	CTD	2.09E-09	4.57E-06	5	71
19	43	Acetylcysteine	CTD	2.23E-09	4.77E-06	9	780
20	48	Micheliolide	STITCH	3.20E-09	6.01E-06	6	175
21	49	Rosiglitazone	CTD	3.21E-09	6.01E-06	11	1571
22	54	Chebulagic Acid	CTD	3.88E-05	7.18E-07	3	5
23	56	Atorvastatin Calcium	CTD	4.35E-05	7.63E-07	6	186
24	57	Deferoxamine	CTD	4.35E-05	7.63E-07	6	186
25	61	Simvastatin	CTD	5.86E-09	9.06E-07	8	581

**Table 5.** Drugs Targeting Dry-AMD Hub Genes Predicted by Toppgene Database

Filtered Position	Unfiltered Position	Name	Source	*P* Value	*Q* Value FDR B&H	Hit Count in Query List	Hit Count in Genome
1	3	SB 203580	CTD	1.67E-20	8.58E-16	16	388
2	5	Acetylcysteine	STITCH	8.68E-20	2.68E-15	17	546
3	6	Prolinedithiocarbamate	CTD	3.67E-19	9.43E-15	10	59
4	7	Linoleic Acid	CTD	6.87E-19	1.52E-14	11	99
5	13	U 0126	CTD	7.11E-18	7.38E-14	15	444
6	14	Glutathione	CTD	7.17E-18	7.38E-14	14	339
7	15	Thioctic Acid	CTD	1.98E-16	1.22E-12	11	163
8	23	Alpha-Tocopherol	CTD	2.12E-16	1.26E-12	11	164
9	25	Losartan	CTD	2.27E-16	1.29E-12	11	165
10	26	Mevalonic Acid	CTD	2.87E-16	1.53E-12	10	111
11	27	Capsaicin	CTD	1.15E-15	5.20E-12	14	488
12	29	Luteolin	CTD	1.58E-15	6.97E-12	10	131
13	34	Metformin	CTD	3.10E-15	1.23E-11	13	400
14	39	Aspirin	CTD	3.74E-15	1.41E-11	15	678
15	41	Curcumin	CTD	4.15E-15	1.53E-11	16	851
16	42	Buthionine Sulfoximine	CTD	4.63E-15	1.66E-11	12	305
17	44	Ursolic acid	CTD	4.89E-15	1.71E-11	8	53
18	45	Kaempferol	CTD	5.89E-15	2.02E-11	10	149
19	46	Daidzein	CTD	6.49E-15	2.15E-11	11	223
20	50	Staurosporine	CTD	7.71E-15	2.33E-12	10	153
21	51	Telmisartan	CTD	7.98E-15	2.37E-12	9	97
22	52	Diclofenac	CTD	9.73E-15	2.83E-12	14	570
23	58	Fisetin	CTD	1.40E-14	3.66E-12	8	60
24	59	Rutin	CTD	1.67E-14	4.23E-12	9	105
25	60	Melatonin	CTD	1.68E-14	4.23E-12	11	243

**Table 6.** Drugs Targeting Intermediate-AMD Hub Genes Predicted by Toppgene Database

Filtered Position	Unfiltered Position	Name	Source	*P* Value	*Q* Value FDR B&H	Hit Count in Query List	Hit Count in Genome
1	1	Curcumin	CTD	6.49E-41	1.53E-36	41	851
2	2	Simvastatin	CTD	2.23E-34	2.62E-30	33	581
3	3	SB 203580	CTD	2.13E-33	1.67E-29	29	388
4	5	Acetylcysteine	CTD	1.29E-31	6.07E-28	34	781
5	6	Losartan	CTD	8.16E-31	3.20E-27	22	165
6	7	Ascorbic Acid	CTD	2.87E-30	9.64E-27	31	627
7	8	U 0126	CTD	1.51E-28	4.43E-25	27	444
8	9	Glutathione	CTD	1.85E-28	4.82E-25	25	339
9	11	Enalapril	CTD	2.44E-27	5.21E-24	19	131
10	13	Betamethasone	STITCH	2.51E-26	4.54E-23	36	1342
11	16	Telmisartan	CTD	5.45E-26	8.00E-23	17	97
12	17	Diclofenac	CTD	1.22E-25	1.69E-22	27	570
13	20	Aspirin	CTD	5.31E-25	6.21E-22	28	678
14	22	Paricalcitol	CTD	7.48E-25	7.99E-22	16	87
15	23	Atorvastatin Calcium	CTD	2.58E-24	2.53E-21	19	186
16	24	Pioglitazone	STITCH	3.98E-24	3.74E-21	22	326
17	28	Thioctic Acid	CTD	1.12E-23	9.05E-21	18	163
18	30	Prolinedithiocarbamate	CTD	1.55E-23	1.18E-20	14	59
19	34	Niacin	CTD	3.61E-23	2.36E-20	17	139
20	36	GW 501516	CTD	4.33E-23	2.75E-20	14	63
21	37	Capsaicin	CTD	4.47E-23	2.76E-20	24	488
22	39	Isoproterenol	CTD	1.15E-22	6.73E-20	30	1017
23	44	Metformin	CTD	3.55E-22	1.85E-19	22	400
24	48	Alpha-Tocopherol	CTD	6.68E-22	3.20E-19	17	164
25	50	Apigenin	CTD	9.29E-22	4.28E-19	18	207

**Table 7.** Drugs Targeting Geographic Atrophy Hub Genes Predicted by Toppgene Database

Filtered Position	Unfiltered Position	Name	Source	*P* Value	*Q* Value FDR B&H	Hit Count in Query List	Hit Count in Genome
1	1	Glutathione	CTD	9.16E-25	1.90E-20	41	851
2	2	Acetylcysteine	CTD	1.25E-21	1.29E-17	33	581
3	3	Alpha-Tocopherol	CTD	2.14E-19	1.48E-15	29	388
4	9	Ascorbic Acid	CTD	3.30E-19	1.71E-15	34	781
5	15	Atorvastatin Calcium	CTD	1.18E-18	4.90E-15	22	165
6	16	Kaempferol	CTD	1.60E-18	5.51E-15	31	627
7	19	Nitroglycerin	CTD	3.62E-18	1.07E-14	27	444
8	37	Triamcinolone Acetonide	CTD	7.03E-18	1.82E-14	25	339
9	42	Simvastatin	CTD	1.10E-17	2.54E-14	19	131
10	47	Cyclophosphamide	STITCH	1.46E-17	2.81E-14	36	1342
11	49	U 0126	CTD	1.49E-17	2.81E-14	17	97
12	52	Curcumin	CTD	2.55E-17	4.40E-14	27	570
13	56	Melatonin	CTD	3.06E-17	4.87E-14	28	678
14	57	Ceramide	CTD	8.09E-17	1.20E-13	16	87
15	58	Deferoxamine	CTD	9.12E-17	1.26E-13	19	186
16	61	SB 203580	STITCH	1.81E-16	2.34E-13	22	326
17	62	Cinnamic Aldehyde	CTD	2.00E-16	2.43E-13	18	163
18	64	Rosiglitazone	CTD	2.45E-16	2.81E-13	14	59
19	69	Pimagedine	CTD	3.85E-16	4.01E-13	17	139
20	70	Metformin	CTD	3.87E-16	4.01E-13	25	553
21	71	Capsaicin	CTD	4.27E-16	4.21E-13	14	63
22	72	Mevalonic Acid	CTD	4.84E-16	4.56E-13	24	488
23	73	Nitroprusside	CTD	6.64E-16	5.98E-13	30	1017
24	74	Docosahexaenoic Acids	CTD	7.66E-16	6.61E-13	22	400
25	75	Rifampin	CTD	8.19E-16	6.78E-13	17	164

**Table 8.** Drugs Targeting Combined Intermediate and Dry-AMD Hub Genes Predicted by Toppgene Database

Filtered Position	Unfiltered Position	Name	Source	*P* Value	*Q* Value FDR B&H	Hit Count in Query List	Hit Count in Genome
1	1	Curcumin	CTD	2.77E-48	7.28E-44	50	851
2	2	SB 203580	CTD	9.80E-46	1.29E-41	39	388
3	3	Acetylcysteine	CTD	2.00E-42	1.76E-38	45	781
4	4	Glutathione	CTD	3.57E-41	2.34E-37	35	339
5	5	U 0126	CTD	3.80E-40	2.00E-36	37	444
6	8	Simvastatin	CTD	2.80E-37	9.19E-34	38	581
7	9	Losartan	CTD	3.90E-37	1.14E-33	27	165
8	15	Ascorbic Acid	CTD	1.41E-34	2.47E-31	37	627
9	18	Alpha-Tocopherol	CTD	1.57E-33	2.29E-30	25	164
10	19	Aspirin	CTD	2.46E-33	3.41E-30	37	678
11	20	Diclofenac	CTD	3.52E-33	4.63E-30	35	570
12	22	Capsaicin	CTD	1.49E-32	1.78E-29	33	488
13	24	Betamethasone	STITCH	4.45E-32	4.73E-29	45	1342
14	27	Thioctic Acid	CTD	8.48E-32	8.25E-29	24	163
15	31	Enalapril	CTD	1.72E-30	1.46E-27	22	131
16	32	Atorvastatin Calcium	CTD	2.38E-30	1.96E-27	24	186
17	35	Telmisartan	CTD	1.11E-29	8.36E-27	20	97
18	36	Linoleic Acid	CTD	1.74E-29	1.27E-26	20	99
19	37	Metformin	CTD	2.46E-29	1.75E-26	29	400
20	39	Kaempferol	CTD	3.52E-29	4.21E-13	14	63
21	41	Melatonin	CTD	4.52E-29	4.56E-13	24	488
22	47	Niacin	CTD	4.37E-28	5.98E-13	30	1017
23	49	Mangiferin	CTD	8.22E-28	6.61E-13	22	400
24	56	Rutin	CTD	4.82E-27	6.78E-13	17	164
25	57	Rosiglitazone	CTD	7.38E-27	3.40E-24	43	1571

**Table 9.** Drugs Targeting Combined Intermediate, Dry- AMD, and Geographic Atrophy Hub Genes Predicted by Toppgene Database

Filtered Position	Unfiltered Position	Name	Source	*P* Value	*Q* Value FDR B&H	Hit Count in Query List	Hit Count in Genome
1	1	Curcumin	CTD	3.21E-48	9.46E-44	57	851
2	2	Glutathione	CTD	1.56E-47	2.30E-43	43	339
3	3	Acetylcysteine	CTD	9.02E-45	8.85E-41	53	781
4	4	SB 203580	CTD	7.16E-42	5.27E-38	41	388
5	5	U 0126	CTD	1.96E-39	1.16E-35	41	444
6	7	Simvastatin	CTD	1.12E-38	4.70E-35	44	581
7	8	Ascorbic Acid	CTD	1.42E-38	5.24E-35	45	627
8	10	Alpha-Tocopherol	CTD	6.86E-38	2.02E-34	30	164
9	17	Losartan	CTD	4.43E-36	7.67E-33	29	165
10	24	Atorvastatin Calcium	CTD	1.79E-34	2.20E-31	29	186
11	29	Capsaicin	CTD	1.27E-33	1.29E-30	38	488
12	32	Thioctic Acid	CTD	7.50E-33	6.90E-30	27	163
13	33	Melatonin	CTD	1.74E-32	1.55E-29	30	243
14	35	Kaempferol	CTD	2.86E-32	2.41E-29	26	149
15	36	Aspirin	CTD	5.71E-32	4.60E-29	41	678
16	39	Diclofenac	CTD	4.27E-31	3.22E-28	38	570
17	41	Isoproterenol	CTD	1.48E-30	1.06E-27	46	1017
18	42	Enalapril	CTD	2.33E-30	1.63E-27	24	131
19	46	Metformin	CTD	6.52E-30	4.13E-27	33	400
20	47	Apigenin	CTD	6.60E-30	4.13E-27	27	207
21	48	Linoleic Acid	CTD	6.95E-30	4.26E-27	22	99
22	49	Docosahexaenoic Acids	CTD	1.08E-29	6.49E-27	25	161
23	55	Betamethasone	STITCH	1.86E-28	9.94E-26	49	1342
24	56	Rosiglitazone	CTD	2.36E-28	1.24E-25	52	1571
25	57	Telmisartan	CTD	2.72E-28	1.40E-25	21	97

The genes, drugs they correlate with, and detailed statistics (*P* value, *q* value FDR B&H, hit in genome, and the genes that each drug they correlate with) for AMD, each subtype and combined subtypes are available in Supplementary Table S1. This data will be made publicly available when the manuscript is accepted for publication.

### Different Drug Classes are Beneficial for Different Sub-Types of AMD

Separated networks were generated using the chemicals and genes specific to the sub-type of AMD disease. In addition to AMD, there were 6 additional AMD subgroups used in this analysis including wet AMD, dry AMD, intermediate AMD, GA, intermediate and dry AMD, and intermediate AMD/dry AMD/GA. The number of drugs- genes interactions and the density of the network are shown in Table 2. The drugs and the category they relate to are available in Supplementary Table S2. This data will be made publicly available when the manuscript is accepted for publication.

We found multiple drug classes and nutrients, for instance, metformin and statins, with well-known pharmacodynamics and safety profiles that could be further investigated and prove efficacious for AMD patients. Curcumin, a flavonoid polyphenol, was identified as the compound with the most significant drug-gene interactions among all AMD-affiliated genes. Among the subtype analysis, again curcumin was identified as the most statistically significant compound for dry AMD -both the intermediate form and GA (Tables 8 and 9); for wet AMD, metformin had the strongest association with risk-genes (Table 4). Of note, several of these compounds identified as top targets in our study such as curcumin, metformin, atorvastatin, and antioxidant formularies are currently under clinical evaluation for the treatment of AMD (Table 5).

Genes from the comprehensive AMD list were analyzed and visualized with Cytoscape, which identified several gene pathway networks involved in AMD. The top networks included “ACE-RAGE signaling pathway in diabetic complications,” “fluid shear stress and atherosclerosis,” “HIF-1 signaling pathway,” “TNF signaling pathway,” “VEGF-VEGFR2 signaling pathway,” and “Insulin resistance–Homo sapiens.” In this network, the NFKB1 gene encoding for Nuclear Factor Kappa B Subunit 1 showed the highest degree value. NFKB1 is a transcription factor found nearly ubiquitously in all cell types that's stimulated by inflammation and stress. Other genes with high degree values and closeness include AKT2, NOS3, tumor necrosis factor, EDN1, VEGF, and MAPK8, which are genes involved in proliferation, growth, and angiogenesis (reference). Of note, these genes correspond to the GO terms “positive regulation of angiogenesis,” lipopolysaccharide-mediated signaling pathway,” and “glucose metabolic process,” which support the identification of pharmacological agents like lipid-lowering agents and antidiabetic medications in our drug-gene pathways as possible pharmaceutical modulators for AMD.

## Discussion

Biological systems are complex with multiple closely intertwined elements, and the disease process reflects disturbances in multiple elements of a system simultaneously. Network medicine offers a platform to systematically explore overlapping biological relationships to reveal molecular connections between apparently distinct pathways. Computational approaches to predict drug-target interactions are easier to perform in comparison to traditional experimental assays. Additionally, recent advances in multiomics have expedited the generation of large-scale, biological networks, which offer a novel method to study heterogeneous disease processes, like AMD. Attempts to understand AMD biology by reductionist methods (i.e., investigations that define biologic systems in terms of their smallest entity] have been only marginally successful as AMD has a heterogeneous and multifactorial pathogenesis. Using systems medicine and big-data are suggested as a solution to overcome the problem to synthesize input from multiple data sources at one time.^16^ In this study, we used the network pharmacology approach to visualize connections from known genes to predict the possible novel drug candidates for AMD at different stages of the disease process. We identified the risk-genes that are involved in AMD, wet AMD, dry AMD, GA, and intermediate AMD, respectively (Supplementary Table S1). Moreover, we combined the intermediate and dry AMD genes and intermediate AMD, dry AMD, and GA genes and analyzed these groups in addition to the above mentioned subtypes. We combined the genes involved in the select groups to better capture genes that play a role in distinct subtypes of AMD. The stepwise pathogenesis of AMD is not clearly understood, and some investigations suggest that wet and dry AMD are distinct disease forms because of their dramatically different response to anti-VEGF drugs.^3^ From these compiled gene lists, a predictive analysis to identify possible drugs that can target the hub genes for each group is done. In contrast to the aforementioned reductionist study methods, our method takes a holistic approach by aggregating multiple networks of interacting molecular and cellular components. This integrative approach is perhaps a closer approximation of the disease process in humans.^16^

Drug discovery using a system biology approach has been undertaken with various pathologies in the past. The study most comparable to our own regarding method and objective was conducted by Platania et al.^26^ in 2018 on diabetic retinopathy (DR), another progressive retinal disease leading to gradual vision loss. Our studies attempt to identify target genes and novel drugs for complex ocular pathologies with a paucity of approved drugs indicated only for a subset of the advanced disease form. Although the DR study used transcriptomics data obtained from the Gene Expression Omnibus Dataset repository to build gene-pathway and drug-gene networks for their analysis, we used the National Center for Biotechnology Information database, which encompasses sources from animal models, human retina, and genotypes studies to identify AMD target genes for our drug-gene network. The differences in approach, however, create an avenue of amalgamating the two methods to identify additional gene and drug targets for both DR and AMD, and this could enhance the complexity and generalizability of the drug-gene networks for both pathologies. Furthermore, the approach of stratifying a disease process into different stages as done in our study could elucidate targets for DR throughout its progression at alternate time points.

Our analysis identified numerous biologically active chemicals and molecules capable of targeting the genes involved in different subtypes of AMD. This also includes Food and Drug Administration (FDA)–approved drugs that could be subsequently repurposed for AMD patients. Many epidemiologic studies associate AMD with cardiovascular diseases because of shared risk factors for both diseases such as dyslipidemia, hypertension,^46^ age over 55, smoking,^47^^,^^48^ genetic predisposition, and postmenopausal state.^49^ The statically significant molecules and drugs enriched by our analysis are either drug classes effective for diseases known to be associated with risk for AMD, like cardiovascular diseases and diabetes, or affect crucial biologic pathways or stressors implicated in AMD pathogenesis like oxidative stress and angiogenesis. A few completely novel chemicals with well-established safety profiles that can be used as therapeutic intervention were also identified. The validity of our method is strengthened by the over-representation of antioxidant AREDS compounds such as ascorbic acid (vitamin C) and alpha-tocopherol (vitamin E) enriched in dry and intermediate AMD, and GA (Tables 7 and 8). Today, AREDS supplements are the only intervention demonstrating a reduced risk of progression to advanced wet AMD; thus we can deduce that other molecules determined by this enrichment analysis could potentially also be of value for further investigation in AMD patients in further preclinical and clinical studies.^8^^,^^9^

The success of the original antioxidant micronutrient AREDS study sparked an interest in antioxidant therapies and nutritional modulation for AMD. Numerous antioxidants including flavonoids, glutathione, and linoleic acid strongly correlate with risk-genes for all AMD subtypes (Tables 3^4^,^5^,^6^,^7^–8, 10). Oxidative stress secondary to increased reactive oxygen species (ROS) is strongly associated with AMD.^50^ ROS increases in retinal pigment epithelium (RPE) secondary to either smoking or genetic variants predispose to damage by ROS. Genetic variants in oxidative stress-related genes, in particular, MTND2*LHON-4917G, NADH enzyme subunits, SOD2, and PPARGC1A are associated with an increased risk of AMD.^51^^–^^53^ However, antioxidant drugs historically had limited success because of challenges with the bioavailability of oral drugs in the ocular tissue and secondly, only a small amount of antioxidants reach the mitochondrion which is responsible for ROS generation.^16^^,^^51^^,^^52^ Nevertheless, the use of antioxidants to prevent and support the present pharmacological treatments is still being vigorously pursued as novel methods of drug delivery to the eye such as nanoformulations become available.^54^ However, additional studies are needed to define the antioxidant class and formulations beneficial for AMD.

**Table 10.** Toppgene Uses Functional Enrichment of Input Gene List Based on Pharmacome (Drug–Gene Associations) Based on the Sources Listed Below

Annotations	77,146
Broad Institute CMAP Down	6100
Broad Institute CMAP Up	6100
CTD	12,437
Drug Bank	3803
STITCH	4870

The number of annotations from each database are also specified.

Curcumin, a nutritional antioxidant, is the most over-represented molecule associated with gene sets of all AMD, wet AMD, and combined intermediate, dry, and GA (Fig. 2, Fig. 3, Fig. 8). Although curcumin is described as a pan-assay interference compound, a chemical substance that can appear as a false positive in high-throughput screens, the role of curcumin in the eye, especially the retina, has been described for years.^44^^,^^55^ Early animal studies demonstrate that curcumin can ameliorate light-induced retinal degeneration in animal models by inhibiting NF-kappa B activation and improving cellular viability by decreasing apoptosis and oxidative stress in the RPE.^56^^–^^62^ The problem with curcumin, as with all other nutritional drugs, remains with bioavailability and drug delivery. Although, a recent prodrug approach has successfully used curcumin diethyl disuccinate to protect RPE cells from oxidative stress–induced death and to decrease H_2_O_2_-induced ROS production.^63^ Furthermore, novel formulations of curcumin such as Norflo (curcumin-phosphatidylcholine) have proven effective in clinical trials for eye pathologies such as uveitis and central serous chorioretinopathy, and curcumin formulations with superior retinal bioavailability are also available using vehicles such as the polyvinylpyrrolidone-hydrophilic carrier.^61^^,^^64^^–^^66^ Additional studies not only with drug delivery systems but also mathematical relationships between chemical structures and biological activities are needed to uncover the precise formulation of the antioxidants and nutrients that are beneficial for AMD.

Our findings also revealed several FDA-approved drugs that could be repurposed for AMD such as metformin. In our analysis, metformin strongly correlated with risk genes of all subtypes of AMD both dry and wet among thousands of compounds appearing as number one for wet AMD and among top 20 for all dry AMD subtypes. Recent investigations by our team and others support these findings by demonstrating that metformin is associated with decreased risk of developing AMD after adjusting for age, gender, and comorbidities.^30^^,^^67^^–^^69^ Metformin is the most commonly used drug for type II diabetes that has been shown to have versatile protective properties for other aging-related conditions such as cardiovascular diseases, dementia, and some carcinomas.^70^^–^^72^ Connection between AMD and diabetes has been proposed through a unifying mechanism in inflammation and breakdown of the blood-brain barrier^73^; therefore it is hypothesized that antidiabetic drugs like metformin may have a preventive and therapeutic role in AMD. Metformin inhibits development of diabetic retinopathy by inducing alternative splicing of VEGF-A in animal models of diabetic retinopathy.^74^ A similar pathway decreasing progressive angiogenesis via decreasing VEGF in wet AMD can be conjectured; however, the drug's preventative role and response to dry AMD cannot be easily explained by previous works. Nonetheless, our findings indicate that metformin can be beneficial for dry AMD patients by its strong correlation to *PPARGC1*, metalloproteinases (*MMP7, MMP9* MMP2), and IL-10 in addition to VEGF (Supplementary Table S1). Ongoing nonrandomized clinical trials currently in phase II using metformin to decrease the progression of geographic atrophy in nondiabetic patients with AMD could provide more insight for this association between metformin and dry AMD.^75^ Our results also demonstrate that thiazolidinediones such as rosiglitazone and pioglitazone, another group of antidiabetic agents, strongly correlate with AMD risk-genes. Pioglitazone showed a strong anti-inflammatory effect in laser-induced choroidal neovascular lesion in initial investigations in mouse models and retinal cell lines.^76^ The precise role of antidiabetic agents in translational, preclinical, and clinical studies are needed to explore their potential role as part of novel therapeutic strategies for AMD.

Lipids play a major role in drusenogenesis. They comprise more than 40% of drusen volume and polymorphisms found in lipid-related genes are associated with elevated AMD risk.^77^^,^^78^ Within our results, we identified several available forms of statins and fibrates—drug classes that target lipid metabolism—to have a statistically significant association with AMD risk-genes. The RPE ingests lipoproteins from circulation and accumulates cholesterol by phagocytosing photoreceptors; and, in the event of lipoproteinemia, the RPE consumes the surplus of lipoproteins and becomes overloaded with cholesterol.^79^ Mechanistically, the use of statins, HMG-CoA reductase inhibitors that suppress cholesterol synthesis, has been speculated to halt AMD progression by lowering lipid levels.^80^ One recent clinical trial reported vision gain with regression of drusen deposits in dry AMD patients that received a high dose of atorvastatin.^29^ Our data further support evidence that FDA-approved lipid-lowering therapies may have the potential in halting the progression of the disease or restoring functionality in AMD patients. Both retrospective large scale studies on AMD incidences in patients taking such medications and eventually moving to larger clinical trials are needed to discern the precise effect of these drugs in vivo*.*

Other cardiac drugs apart from lipid-lowering agents also feature prominently in this analysis. These drugs belong to various categories including antihypertensive, antiarrhythmic, and antiplatelet agents, though the links between cardiovascular disease and AMD are difficult to delineate as the patients with cardiovascular disease also have multiple problems simultaneously.^48^ Current guidelines suggest that the overall benefits of aspirin use on decreasing the risk of cardiovascular incidents far outweigh the harm of aspirin use associated with AMD progression.^81^ In a recent multivariable analysis of 1011 study eyes without baseline GA, systemic medications including cholinesterase inhibitor, ACE inhibitors, calcium channel blockers, beta-blockers, diuretics, aspirin, steroids, statins, hormone replacement therapy, antacids, and drugs targeting G protein-coupled receptors, were not associated with GA incidence in the study eye (all adjusted hazard ratios ≤1.86, *P* ≥ 0.18).^82^ However, calcium channel blockers were associated with a higher GA growth rate, and calcium channel blockers like nifedipine show a strong correlation with genes for wet-AMD identified by our analysis.^82^ The results from the multivariable analysis should be interpreted with caution, as the small risk from CCBs may be attributed to confounding variables and these results need to be validated by more preclinical studies and prospective clinical trials.

Other classes of drugs demonstrated by this analysis include MAPK inhibitors and MEK 1/2 inhibitors. Both the novel MAPK and MEK 1/2 drugs can cause serious ocular toxicities; including retinal vein occlusion, uveitis, and retinal pigment epithelial detachment which limit their use in the current state.^83^ However, these drugs are attractive for AMD because they target the MAPK pathway, which closely intertwines with VEGF and HIF-1 signaling. The work on these drugs is in its infancy, and additional experiments to modify the structure and toxicities spectrum of these agents are needed.

### Limitations

It is difficult to produce a faithful model for AMD as the disease has very heterogeneous etiology. The complex interplay between genetics, diet, lifestyle, microbiome, and inflammation is involved in the disease process. Because we only consider risk genes to predict drugs, there are certain limitations in the approach taken in this article. First, datasets such as STITCH and CTD are likely to have inherent errors, such as false-positives or chemical-gene associations resulting from activity of molecules downstream of the original chemical interactions making it challenging to determine the relative value of each chemical-gene association. Second, genomics-based predictions rely on previously published literature to identify drug-gene interactions; therefore biases arise from the proportion of literature present for a certain disease. Consequently, highly investigated diseases, for example, cancer and nervous system disorders will have more reported chemical and gene associations compared to their lesser studied counterparts. A similar bias is present with over representation of well-studied drugs having disproportionate chemical-disease associations in contrast to little known drugs that are not accurately captured in enrichment analyses because of lack of relevant gene-disease studies. Third, we did not account for positive (protective variant) or negative (risk variant) effects of the genes. In vitro/preclinical and clinical studies need to be performed to elucidate the precise effect of the drug on ocular tissue. Fourth, the initial gene list also includes data from animal models and transformed cell lines; therefore some reported drug-gene interactions may not be physiologically relevant in humans.

A systems biology approach can identify potential drug candidates among existing compounds that could be repurposed for AMD.

## Conclusion

Overall, our analysis uncovers potentially useful drugs and molecules for different AMD subtypes. Relatively similar drug classes for different AMD subtypes like antidiabetics, statins and antioxidants have strong associations with AMD genes for all different types of AMD, suggesting that similar drug classes can be repurposed/proposed for all subtypes of AMD. Despite potential shortcomings, this model of using advanced bioinformatics tools and drug-gene association studies and expanding to potentially applying an integrated systems biology approach including metabolomics, proteomics, and microbiomics data could assist in formulating new hypotheses, identifying interesting or novel domains of investigations, improving our comprehension about both disease etiology and therapeutic targets, and ultimately aiding in planning future clinical trials. We found multiple drug classes and nutrients, for instance, metformin and statins, with well-known pharmacodynamics and safety profiles that could be further investigated and potentially prove efficacious for AMD patients. Because this analysis is an objective and unbiased manner of predicting therapeutic targets, this compilation can serve as a basis for future basic science and translational studies for AMD scientists.

As more studies with multi-omics data from human AMD patients and their association with clinical phenotype and genetic risk become available, the precision of such predictive mathematical computational models of drug-targets-association will likely increase. This study highlights the need for computational and bioinformatics approaches to advance the understanding of complex diseases like AMD and we believe that these methods can be used in future for other complex multifactorial incurable diseases.

## Supplementary Material

Supplement 1

Supplement 2
